# Photopolymerizable Ionogel with Healable Properties Based on Dioxaborolane Vitrimer Chemistry

**DOI:** 10.3390/gels8060381

**Published:** 2022-06-15

**Authors:** Fengdi Li, Giao T. M. Nguyen, Cédric Vancaeyzeele, Frédéric Vidal, Cédric Plesse

**Affiliations:** Laboratory of Physicochemistry of Polymers and Interfaces, CY Cergy Paris Université, 5 Mail Gay Lussac, 95000 Neuville sur Oise, France; fengdi.li@cyu.fr (F.L.); tran-minh-giao.nguyen@cyu.fr (G.T.M.N.); cedric.vancaeyzeele@cyu.fr (C.V.); frederic.vidal@cyu.fr (F.V.)

**Keywords:** ionogel, vitrimer, polythioether, solid electrolyte, self-healing

## Abstract

Ionogels are solid polymer gel networks loaded with ionic liquid (IL) percolating throughout each other, giving rise to ionically conducting solid electrolytes. They combine the mechanical properties of polymer networks with the ionic conductivity, non-volatility, and non-flammability of ILs. In the frame of their applications in electrochemical-based flexible electronics, ionogels are usually subjected to repeated deformation, making them susceptible to damage. It appears critical to devise a simple and effective strategy to improve their durability and lifespan by imparting them with healing ability through vitrimer chemistry. In this work, we report the original in situ synthesis of polythioether (PTE)-based vitrimer ionogels using fast photopolymerization through thiol-acrylate Michael addition. PTE-based vitrimer was prepared with a constant amount of the trithiol crosslinker and varied proportions of static dithiol spacers and dynamic chain extender BDB containing dynamic exchangeable boronic ester groups. The dynamic ionogels were prepared using 50 wt% of either 1-Ethyl-3-methylimidazolium bis(trifluoromethylsulfonyl) imide or 1-Ethyl-3-methylimidazolium trifluoromethanesulfonate, both of which were selected for their high ionic conductivity. They are completely amorphous (*T_g_* below −30 °C), suggesting they can be used at low temperatures. They are stretchable with an elongation at break around 60%, soft with Young’s modulus between 0.4 and 0.6 MPa, and they have high ionic conductivities for solid state electrolytes in the order of 10^−4^ S·cm^−1^ at room temperature. They display dynamic properties typical of the vitrimer network, such as stress relaxation and healing, retained despite the large quantity of IL. The design concept illustrated in this work further enlarges the library of vitrimer ionogels and could potentially open a new path for the development of more sustainable, flexible electrochemical-based electronics with extended service life through repair or reprocessing.

## 1. Introduction

Ionogels belong to the general class of polymer gels, which may be regarded as solid and liquid phases that percolate throughout each other. They are termed ionically conducting membranes when the ionic liquid (IL) is loaded within a polymer gel network. Such materials are more precisely achieved by in situ polymerization of the gel network in the presence of ILs or by swelling a polymer network with ILs [1]. Ionogels combine the mechanical properties of crosslinked polymer networks with the ionic conductivity, non-volatility, and non-flammability of ILs [1,2,3,4,5,6]. The remarkable physicochemical properties of ionogels make them promising candidates for applications in flexible electronics. Our group has previously reported the synthesis of such ionogels based on polythioether (PTE) networks and ILs for use as solid electrolytes in electrochemical devices [1,4]. More precisely, these ionogels were obtained from the reaction of multifunctional thiols on diacrylate using thiol-ene Michael addition chemistry in the presence of ILs. The thiol-ene Michael addition is a reaction that involves a base- or nucleophile-catalyzed addition of a thiolate anion to electron-deficient alkenes such as maleimides, vinyl sulfones, acrylates, and methacrylates [7]. The sole difference between base- and nucleophile-catalyzed reactions lies in the way the thiolate anion is generated. Classified as a ‘click’ chemistry, the thiol-ene Michael addition is rapid, highly efficient, generates no by-product, and exhibits a nearly ideal 1: 1 stoichiometric reactivity [8,9]. Owing to the stoichiometric reactivity of the thiol-ene Michael addition, a fine-tuning of the surface functionality and mechanical properties of ionogels was possible [1].

In the frame of their applications in electrochemical-based flexible electronics, ionogels are usually subjected to repeated deformation, making them susceptible to damage. Thus, it is critical to devise a simple and effective strategy to improve their durability and lifespan. Imparting ionogels with healing ability seems to be a promising approach because of their capability to repair mechanically induced damage. Hydrogen bonds, ionic bonds, and metal–ligand coordination have all been explored to develop healable ionogels [10,11,12,13]. Even though ionic and hydrogen bonds have been shown to demonstrate effective self-healing properties, these physical networks are generally vulnerable to heat, water, and other polar solvents. Another strategy to endow materials with self-healing capability is to introduce reversible covalent bonds within a chemically crosslinked network. The fabrication of dynamic reversible polymer networks has become a popular strategy, notably by introducing exchangeable chemical bonds into polymer networks, which are known as covalent adaptable networks (CANs). CANs are further divided into dissociative and associative mechanisms based on the intrinsic mechanism of the bond-exchange reaction [14]. In dissociative CANs, bonds are first broken and then reformed in response to external stimuli, such as heat or light [14,15,16]. Healable ionogels have been reported using dissociative bond-exchange reactions [17,18]. However, dissociative CANs allow topological rearrangements due to a sudden viscosity drop and uncrosslinking, which is a drawback in applications requiring stability toward solvents, easy shaping, and a welding process. CANs that rely on an associative bond exchange reaction are characterized by a constant crosslink density [19]. As the bond cleavage is accompanied by the simultaneous formation of a new crosslink, such systems can change their topology with no loss of connectivity, making such networks permanent and insoluble. More specifically, in 2011, the term ‘vitrimers’ was introduced by Leibler et al. for thermally activated associative CANs [20]. A few groups have reported vitrimer ionogels. Healable and reprocessable gelatine ionogels based on the reversible exchange of imine bonds have been designed for flexible supercapacitors [21]. Xu et al. reported polyurethane (PU) ionogels that can be readily healed at room temperature and restore their original performance owing to the dynamic boronic ester crosslinker used in the polymer network [22]. The exchange reactions between boronic ester linkages do not generally need a catalyst, initiator, or elevated temperature, and the activation energy of this exchange is relatively low. Another team also showcased healable and recyclable boronic ester-based ionogels using 1-butyl-3-methylimidazolium tetrafluoro-borate [23].

**Figure 1:**
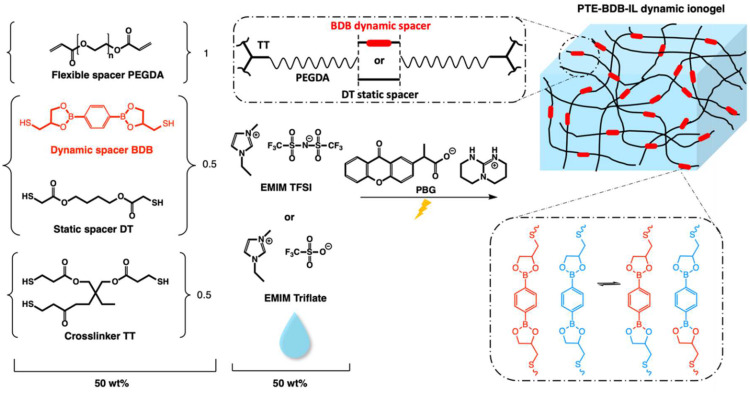
Chemical composition and illustration of the PTE-BDB-IL dynamic ionogels based on boronic ester exchange reaction. We used a 1:1 stoichiometric ratio of acrylate and thiols (1.0 diacrylate:0.5 dithiol:0.5 trithiol). Samples were prepared in different proportions between the flexible spacer DT and the boronic ester dynamic spacer BDB, which is capable of a fast bond exchange reaction. Fifty wt% of either EMIM TFSI or EMIM Triflate compared to the total weight is used to prepare PTE-BDB-IL dynamic ionogels.

The goal of this work was to synthesize an original ionically conducting polythioether-based vitrimer based on ionogels using fast photopolymerization (Figure 1). The ionogels’ mechanical properties can be fine-tuned thanks to the 1:1 stoichiometric reactivity of thiol-acrylate Michael addition. Either 1-Ethyl-3-methylimidazolium bis(trifluoromethylsulfonyl)imide (EMIM TFSI) or 1-Ethyl-3-methylimidazolium trifluoromethanesulfonate (EMIM Triflate) was chosen as ionic liquid in these ionogels due to their high ionic conductivity. While keeping the 1:1 stoichiometric ratio between thiol and acrylate groups, and the amount of the crosslinker constant, the choice of the thiol spacers is varied between dynamic dithiol-containing boronic ester spacer and non-dynamic dithiol spacer to wisely control the dynamic properties. The thermal properties, mechanical properties, and ionic conducting behavior of these ionogels were studied to highlight their wide working temperature range, good mechanical properties, and polymer electrolyte propensity. The dynamic properties of these solid-state electrolytes were examined with healing and stress relaxation experiments. This work further enlarges the library of vitrimer ionogels and provides one simple and effective method to develop healable ionogels with satisfying ionic conductivity.

## 2. Results and Discussion

We have previously reported the preparation and characterization of PTE-based ionogels using thiol-ene Michael addition between a mixture of different thiol and acrylate functional groups [1]. The resulting soft and stretchable ionogels can withstand repeated use and considerably large deformation without failure, making them potential candidates for use in the development of wearable and stretchable electronic devices. Therefore, in this study, similar compositions of ionogel were used as a starting point for the preparation of PTE-based dynamic ionogels. That is, using poly(ethylene glycol) diacrylate (PEGDA) as an electron-deficient partner, 1,4-Butanediol Bis(thioglycolate) (dithiol, DT), trimethylolpropane tris(3-mercoptopropianate) (trithiol, TT) as a thiol partner with their a functional group molar ratio of 100:50:50 in the presence of 50 wt% ionic liquid relative to the total mixture weight.

### 2.1. Synthesis and Characterization of PTE-BDB Dynamic Networks

In this study, we use a novel dithiol bearing boronic ester groups (2,2′-(1,4-Phenylene)-bis[4-mercaptan-1,3,2-dioxaborolane], BDB, Figure 1) to introduce exchangeable bonds into our PTE-based ionogel. This dithiol has recently been reported to be used as a dynamic crosslinker with pendant vinyl groups of styrene-butadiene rubber chains via a thermally initiated thiol-ene “click” reaction [24]. In this frame, BDB was synthesized following the protocol reported by Chen et al. [24]. Dynamic PTE-BDB networks were prepared by photopolymerization with the presence of 1 wt% of photobase generator (PBG vs the total weight of precursor mixture). While keeping the 1:1 stoichiometric ratio between thiol and acrylate groups, and the amount of trithiol crosslinker (0.5 molar ratio of thiol groups of TT), the choice of the thiol spacers (0.5 molar ratio, by reactive bonds) is varied between dynamic BDB and static DT to control the dynamic properties. Three systems were prepared: (i) non-vitrimer system PTE-BDB0 with 50 mol% of thiols from the DT static spacer (i.e., 100% of dithiol spacers being static DT); (ii) partial vitrimer system (PTE-BDB25) with 0.25 molar ratio of BDB and 0.25 molar ratio of DT static spacer; (iii) full vitrimer system PTE-BDB50 with 50 mol% of thiols from BDB dynamic spacer (i.e., 100% of dithiol spacer being the dynamic BDB). The chemical structures of all chemicals are illustrated in Figure 1. The composition and properties of all samples are reported in Table 1.

**Figure 2:**
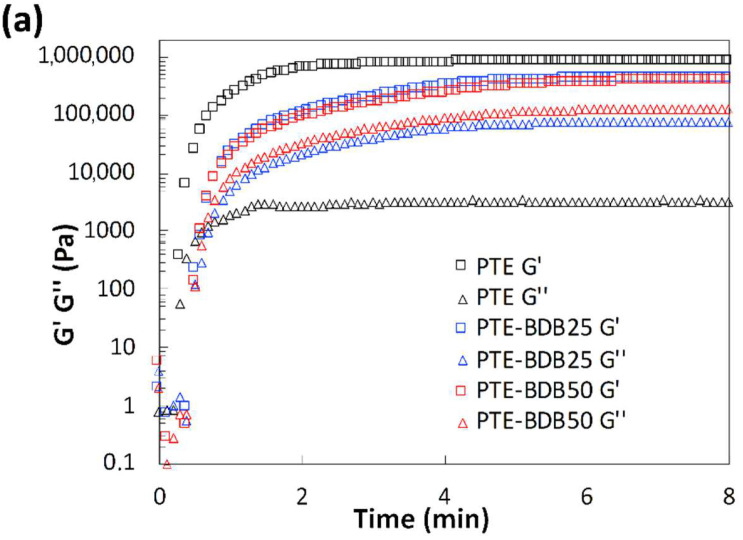

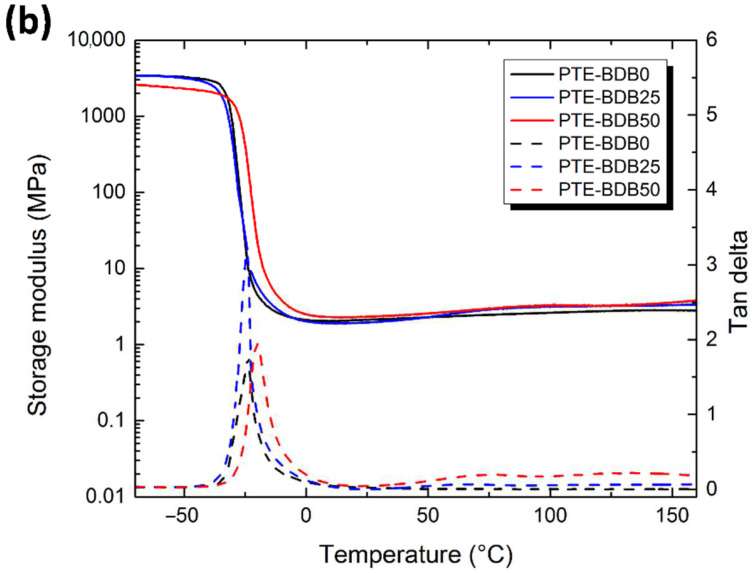
(**a**) Rheological studies of PTE-BDB0, PTE-BDB25, and PTE-BDB50 precursor mixtures during in-situ photopolymerization; (**b**) DMA curves of PTE-BDB series samples.

Rheological studies were carried out on PTE-BDB samples (containing 0 mol%, 25 mol% or 50 mol% of dynamic spacers) by pouring the precursor mixtures into the rheometer followed by an in-situ photopolymerization at 30 °C. The storage modulus (G′) and loss modulus (G″) were recorded as a function of time (Figure 2a). The gel points, where G’ and G″ curves intersected, were reached within 30 s for the PTE-BDB25 and PTE-BDB50 samples and within 20 s for the PTE-BDB0 sample, thanks to the fast thiol-ene Michael addition. PTE-BDB25 and PTE-BDB50 samples reached a G′ plateau of about 400 kPa within 5 min, while PTE-BDB0 reached a G′ plateau of 800 kPa within 3 min. The slower polymerization kinetics indicate that the thiol functional groups of the BDB dynamic spacer are less reactive than the thiol groups of the DT static spacer. This observation is in agreement with the increased steric hindrance of the BDB chain extender, which would slow the chain transfer step of thiol-ene Michael addition [25]. The lower storage modulus could imply less efficient incorporation of the more rigid BDB crosslinker.

The soluble fractions of all samples were extracted in DCM at 60 °C under 100 bar to verify the successful formation of polymer networks. Those of the PTE-BDB25 and PTE-BDB50 samples were 8 wt% and 12.5 wt%, respectively, which were higher than that of the PTE-BDB0 sample without a dynamic spacer (3.9 wt%). BDB accounted for 8 wt% of the total weight of precursors for preparing the PTE-BDB25 sample and 15.7 wt% for the PTE-BDB50 sample. To verify that BDB was successfully incorporated into the polymer networks, extractable contents were examined by ^1^H NMR. The NMR spectrum of the soluble fractions of the PTE-BDB25 and PIL-BDB50 samples can be found in Figures S2 and S3, respectively. Integrations of peaks originating from precursors were examined, and the ratios between PEGDA, BDB, DT, and TT were calculated. PTE-BDB25 and PTE-BDB50 samples were prepared using PEGDA, BDB, DT, and TT precursors with functional group ratios of 100:25:25:50 and 100:50:0:50, respectively. Therefore, in the case of stoichiometric reactivity, the theoretical ratios of PEGDA/BDB/DT/TT extracted should be 6/1.5/1.5/2 for the PTE-BDB25 sample and 6/3/0/2 for the PTE-BDB50 sample. Based on the calculation, residues of all samples after extraction were found to demonstrate ratios close to the theoretical values, even if the proportion of BDB tended to be progressively overrepresented in the extractable fraction with increasing dynamic crosslinker content (6/1.9/1.1/1.7 for PTE-BDB25 and 6/4.9/0/3 for PTE-BDB50). These results indicate that all thiol and acrylate precursors participated in the polymerization successfully but also confirm the less reactive nature of BDB.

Thermal and mechanical properties: Differential scanning calorimetry (DSC), dynamic mechanical analysis (DMA), and tensile strength tests were conducted to study the thermomechanical properties of these materials and to evaluate how dynamic spacer content impacts these properties. All samples were completely amorphous, and no crystallization or melting was observed (Figure S4). Moreover, these materials demonstrate only one glass transition. The onset *T_g_* values are listed in Table 1. There is an evident shift to higher *T_g_* values when the aromatic BDB chain extenders replace the more flexible DT spacers, as previously reported [26,27]. The more rigid BDB moieties restrict the segmental chain mobility of the polymer networks, leading to a stronger glass propensity. Figure 2b shows the storage modulus and tan d versus temperature of all samples with different contents of BDB chain extenders. At low temperatures, the storage modulus of the glassy states of all samples was about 2 GPa, and then decreased remarkably when the samples went through an evident and narrow α relaxation. All curves exhibited only one transition, which is in line with the results obtained by DSC. The values of the relaxation temperature T_α_ values, which correspond to the peak of tan δ versus the temperature curve, are around −23 °C. In addition, the storage moduli of all samples in rubbery states were similar. The influence of BDB spacer loading on the mechanical properties of polymer networks was examined using tensile strength tests (Table 1). By replacing 25 mol% of the DT spacer with the BDB spacer, Young’s modulus increased from 1.2 MPa for the PTE-BDB0 sample to 1.5 MPa for the PTE-BDB25 sample. The Young’s modulus then slightly decreases to 1.3 MPa for the PTE-BDB50 sample, probably due to the higher soluble fraction, which suggests that crosslinking is less advanced compared to PTE-BDB25. The resulting PTE-BDB50 network, in this case, contains more dangling chains and is therefore softer. The elongation at break values of all systems remained similar at around 70%.

Stress relaxation behavior: Owing to bond exchange reactions of vitrimers at elevated temperatures, these materials demonstrate macroscopic flow and stress relaxation enabled by reversible rearrangement of the crosslinked network, without risking permanent loss of material properties [28]. In contrast, conventional thermosets are resistant at elevated temperatures (but below the degradation temperature) to relaxation under an applied strain due to their stable crosslinked networks [29]. To study the exchange dynamics of boronic ester groups, PTE-BDB samples were subjected to stress relaxation experiments at various temperatures by monitoring the decrease in stress over time at a constant strain of 3%.

**Figure 3:**
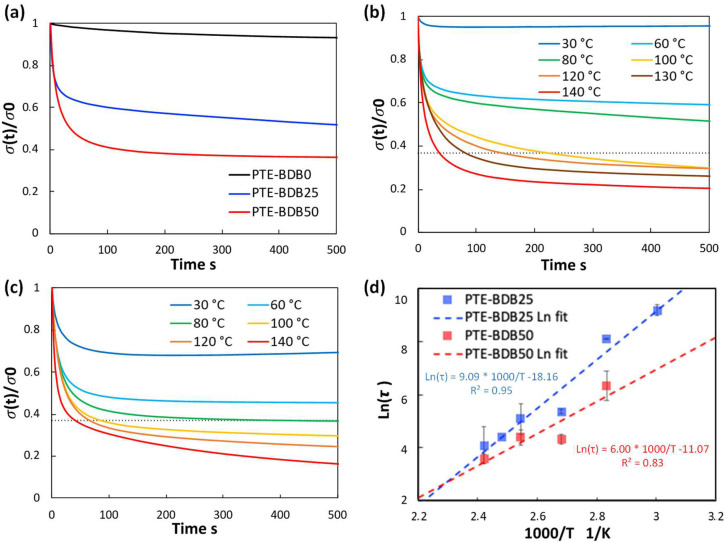
(**a**) Stress relaxation behaviors of PTE-BDB samples with 0, 25, and 50 mol% of BDB dynamic chain extender at 80 °C; (**b**) Stress relaxation curves of PTE-BDB25 sample at different temperatures; (**c**) Stress relaxation curves of PTE-BDB50 sample at different temperatures; (**d**) Arrhenius linear fit of relaxation times of PTE-BDB25 and PTE-BDB50 samples plotted as a function of 1000/T.

Figure 3a compares the relaxation curves of samples containing 0, 25, and 50 mol% of BDB spacers at 80 °C, where the relaxation percentage was plotted as a function of time. PTE-BDB0 sample containing no BDB dynamic spacer relaxed very few percentages of the stress before reaching a plateau, demonstrating the typical elastic response of a thermoset. Upon increasing the BDB content and decreasing the fraction of the permanent network, samples demonstrated stress relaxation behavior, with PTE-BDB25 and PTE-BDB50 relaxed about 50% and 65% of the stress, respectively, within 500 s. These results provide convincing evidence of the exchange reaction between boronic ester groups, allowing network rearrangement in the PTE-BDB samples. Figure 3b,c compare the stress relaxation behavior of the PTE-BDB25 and PTE-BDB50 samples at different temperatures, respectively. At 30 °C, PTE-BDB25 and PTE-BDB50 rapidly relaxed 5% and 30% of the stress, followed by a stress plateau. By increasing the temperature from 30 °C to 140 °C, both the relaxation rate and the eventual relaxation extent of the PTE-BDB25 and PTE-BDB50 samples increased. These results indicate that at low temperatures, the boronic ester exchange is limited and the network rearrangement is almost ‘frozen’. At elevated temperatures, the exchange reaction is activated and demonstrates its temperature-dependent nature as the relaxation rate increases with temperature. At 140 °C, the stress relaxation percentages of PTE-BDB25 and PTE-BDB50 stabilized at 83% and 92%, respectively. Full stress relaxation could not be reached even after 1 h, indicating the probable presence of random non-dynamic TT-PEGDA-TT segments within the polymer networks, leading to materials demonstrating vitrimer-like behaviors [30].

Relaxation time is defined as the time required to relax to 37% (1/e) of initial stress [31]. The relaxation time of the PTE-BDB25 and PTE-BDB50 samples can be found in Table S1. PTE-BDB50 sample with a higher BDB fraction exhibits shorter relation times than the PTE-BDB25 sample at the same temperature. A faster network rearrangement is expected with an increasing number of exchangeable boronic ester linkages. Moreover, the relaxation times exhibited an Arrhenius-like temperature dependence (Figure 3d), indicating the associative exchange mechanism of the boronic ester exchange reaction [24]. Accordingly, the activation energies (*E_a_*) were calculated to be 75.6 kJ·mol^−1^ and 49.9 kJ·mol^−1^ from the slope of the Arrhenius linear fit of PTE-BDB25 and PTE-BDB50, respectively. These values are within the range of 50–90 kJ·mol^−1^ reported in the literature, where the same dioxaborolane groups are used as dynamic chain extenders [27,32]. The more available boronic ester groups existing in the PTE-BDB50 network resulted in faster exchange kinetics and consequently lower activation energy of the viscous flow. As another characteristic key for vitrimer materials, the hypothetical topology freezing temperature (*T_v_*) is conventionally chosen as the temperature at which the viscosity equals 10^12^ Pa.s that describes the liquid-to-solid transition of a glass-forming liquid [14,20]. The hypothetical *T_v_* values can be calculated from the relaxation times using the method described in the experimental section. *T_v_* of PTE-BDB25 and PTE-BDB50 were calculated to be 11.7 °C and −31 °C, respectively (Table S1). Despite the low *T_g_* and *T_v_* values, as indicated by the relaxation experiments, a relaxation stress plateau was observed at 30 °C for both materials, confirming their stable topological behavior at room temperature.

**Figure 4:**
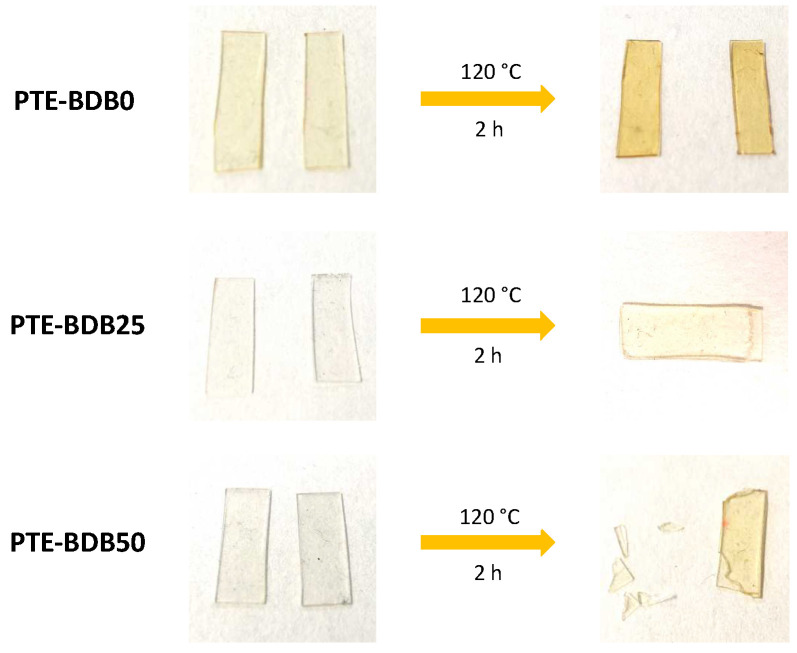
Healing behavior of PTE-BDB samples at 120 °C for 2 h.

Healing behavior: Vitrimers are polymers that can change their topology through dynamic exchange reactions without degenerating the network, maintaining a constant crosslink density [14,20,33,34,35,36]. To examine the dynamic exchange of boronic ester groups, the healing abilities of PTE-BDB series samples were studied by cutting film samples into two pieces and stacking them face-to-face. The films were clipped between two glass plates at 120 °C for 2 h. Pictures of all samples before and after the healing test are shown in Figure 4. After 2 h, the two pieces of PTE-BDB0 could be easily separated, and no healing effect could be observed. In contrast, the two pieces of the PTE-BDB25 and PTE-BDB50 samples could not be separated without breaking them. However, the two film pieces were not fully fused, indicating incomplete healing, which is consistent with the incomplete stress relaxation described earlier. These results proved that boronic ester exchange is directly responsible for the healing behavior of our materials.

### 2.2. Synthesis and Characterization of PTE-BDB-IL Dynamic Ionogels

PTE-BDB25 and PTE-BDB50 samples demonstrated dynamic properties such as stress relaxation and healing thanks to the network reorganization enabled by the exchange reaction of boronic ester bonds. To combine these interesting properties with ionically conducting behavior, ionic liquids were incorporated into these networks. A content of 25 mol% of BDB dynamic spacer was selected because of the higher dimensional stability of the PTE-BDB25 sample. Fifty wt% of either EMIM TFSI or EMIM Triflate was incorporated into the reagent mixture in order to achieve in situ ionogel formation. The chemical formula of the precursors and illustration of the PTE-BDB-IL dynamic ionogels based on the boronic ester exchange reaction are shown in Figure 1. Table 2 compares the compositions and properties of the PTE-BDB-IL ionogels with the PTE-BDB25 sample.

**Figure 5:**
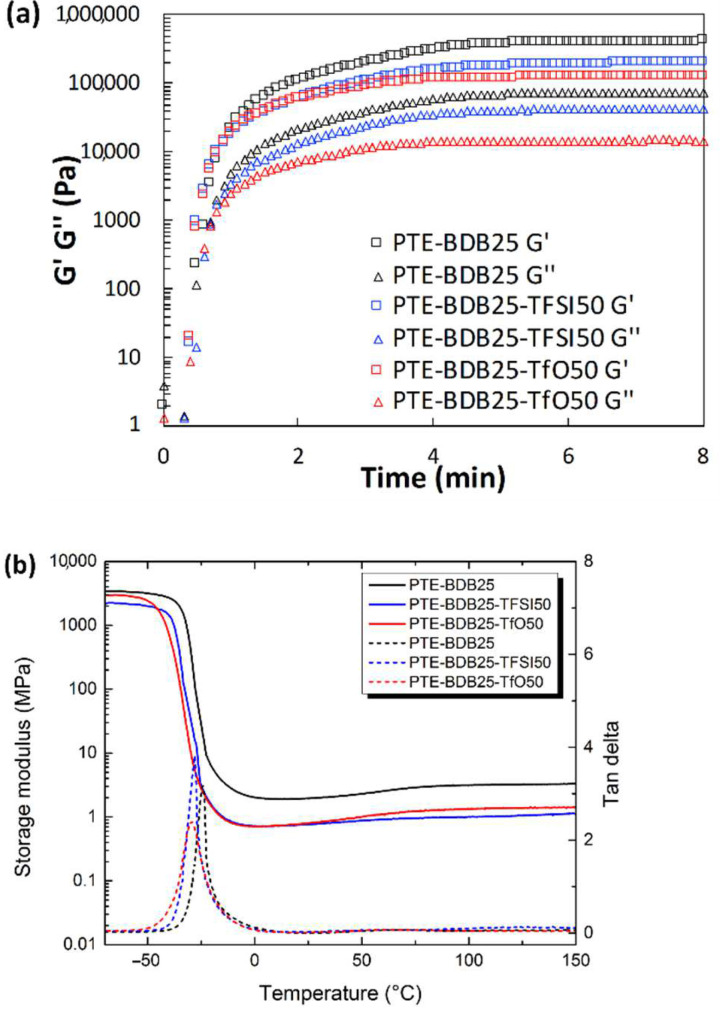
(**a**) Rheological studies of the PTE-BDB25-TFSI50 and PTE-BDB25-TfO50 precursor mixtures during in situ photopolymerization compared with PTE-BDB25 samples; (**b**) DMA tests of PTE-BDB-IL ionogels and PTE-BDB25 sample.

Soluble fractions of PTE-BDB25-TFSI50 and PTE-BDB25-TfO50 TfO50 (i.e., PTE sample containing 25 mol% of BDB and 50 wt% of EMIM Triflate) in DCM were 53.8% and 56.3% respectively. Considering that 50 wt% of ILs were used to prepare the ionogels, these results demonstrated that the polymer networks are well formed. The rheological properties of the PTE-BDB25-TFSI50 (i.e., PTE sample containing 25 mol% of BDB and 50 wt% of EMIM TFSI) and PTE-BDB25 precursor mixtures were monitored during photopolymerization and compared to the PTE-BDB25 sample (Figure 5a). As the liquid precursor solution turned into solid crosslinked ionogel when exposed under UV, the liquid-to-solid transition indicated by the cross-over of the G″ and G′ curves was attributed to the gel point [37,38,39]. The gel points of both samples were reached within 30 s, as in the case of the corresponding single network, demonstrating the fast polymerization kinetics of the thiol-ene Michael addition. G′ plateaus of 200 kPa for the PTE-BDB25-TFSI50 sample and 120 kPa for the PTE-BDB25-TfO50 sample were reached within 5 min. They are lower than the G′ plateau of PTE-BDB25 (400 kPa) prepared in bulk, which indicates material softening in the presence of ILs.

Thermal and mechanical properties of the ionogels: The thermal properties of ionogel samples were studied and compared with the PTE-BDB25 sample (Figure 5b). All samples are completely amorphous materials; no crystallization or melting could be seen in the thermograms, even at temperatures lower than the melting point of the pure ionic liquids (Figure S5). These materials displayed only one glass transition, suggesting that these ionogels can be potentially utilized at temperatures as low as −20 °C. *T_g_* values of −49.4 °C and −51.8 °C were found for PTE-BDB25-TFSI50 and PTE-BDB25-TfO50, respectively, which were lower than that of PTE-BDB25 (−41 °C). Indeed, the plasticizing effect of ILs is responsible for the lower *T_g_* values of ionogels. The storage modulus and tan d versus temperature of all samples are compared in Figure 5b. The addition of ILs resulted in a slightly lower storage modulus at both glassy and rubber states and lower T_α_ values. Tensile tests were conducted on the PTE-BDB25-TFSI50 and PTE-BDB25-TfO50 samples. The values of Young’s modulus and elongation at break are listed in Table 2. Compared to the PTE-BDB25 sample, these ionogels demonstrated lower tensile strengths and similar stretchability to the presence of ILs.

**Figure 6:**
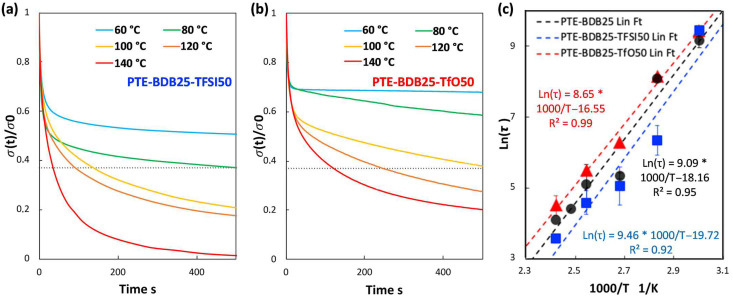
(**a**) Stress relaxation tests of PTE-BDB25-TFSI50 sample at various temperatures; (**b**) The stress relaxation tests of the PTE-BDB25-TfO50 sample at various temperatures; (**c**) Arrhenius linear plot extracted from relaxation times of the (■) PTE-BDB25-TFSI50, (▲) PTE-BDB25-TFSI50, and (●) PTE-BDB25 samples at different temperatures.

Dynamic behavior of the ionogels: PTE-BDB-IL ionogels were also subjected to stress relaxation experiments carried out between 60 and 140 °C to study whether the addition of ILs would modify the dynamic properties enabled by the boronic ester groups of the PTE-BDB25 network. Table S2 compares the stress relaxation behaviors of the PTE-BDB25, PTE-BDB25-TFSI50, and PTE-BDB25-TfO50 samples. Stress relaxation curves of the PTE-BDB25-TFSI50 and PTE-BDB25-TfO50 at different temperatures can be found in Figure 6a,b, respectively. In both cases, the relaxation rate increases with temperature, as the relaxation process is essentially controlled by the thermally activated boronic ester exchange reaction, the rate of which increases with temperature. The relaxation behavior of ionogel is represented as Arrhenius-like temperature dependence in Figure 6c. As for the single networks, *E_a_* and *T_v_* were calculated to be 78.7 kJ·mol^−1^ and 0.3 °C for PTE-BDB25-TFSI50, 72.0 kJ·mol^−1^ and 2.0 °C for PTE-BDB25-TfO50, which were comparable to those values of PTE-BDB25 sample (75.6 kJ·mol^−1^ and 11.7 °C). The similar *E_a_* values indicate that the ILs are spectator compounds in regard to the exchange reaction. However, the behavior of PTE-BDB25-TFSI50 seems to deviate from the expected linear behavior. This can be due to experimental error but also to the presence of TFSI^-^ counterion susceptible to form boron-TFSI adduct responsible for a significant decrease of relaxation times, as previously reported in the presence of LiTFSI salt [40]. The *T_v_* displayed the same 10 °C decrease as observed for the *T_g_*. We can assume that this comes from the already mentioned IL plasticizing effect, which promotes the mobility and rearrangement of the polymer chain at low temperature.

**Figure 7:**
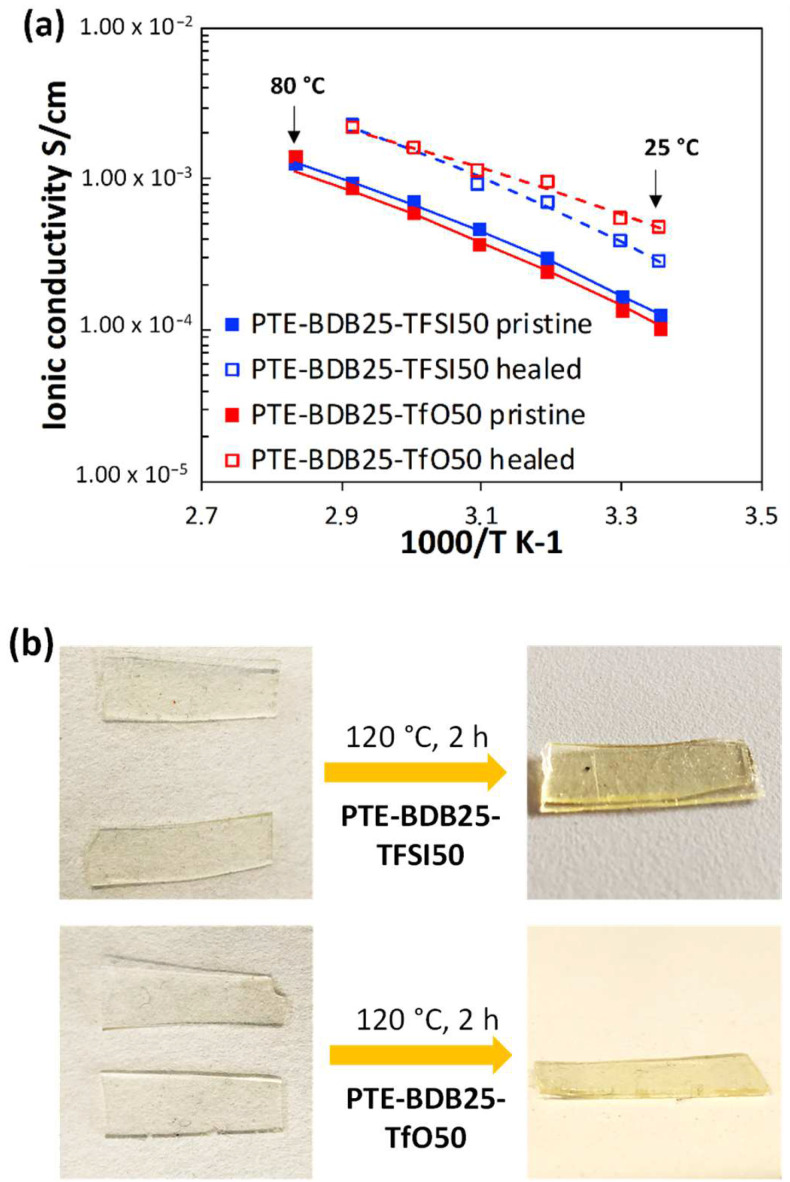
(**a**) Ionic conducting behaviors of PTE-BDB25-TFSI50 and PTE-BDB25-TfO50 samples at different temperatures before and after the healing test at 120 °C for 2 h; (**b**) Pictures of PTE-BDB25-TFSI50 and PTE-BDB25-TfO50 samples before and after healing.

Ionic conducting and healing profiles of the ionogels: After demonstrating that the presence of ILs does not inhibit the boronic ester dynamic exchange. The ionic conductivity behaviors of PTE-BDB-IL ionogels at different temperatures were studied using electrochemical impedance spectroscopy (Figure 7a). At 25 °C, PTE-BDB25-TFSI50 and PTE-BDB25-TfO50 demonstrated ionic conductivities of 1.3 × 10^−4^ S·cm^−1^ and 1.1 × 10^−4^ S·cm^−1^, respectively. Such conductivity has been proven to be satisfying for a wide range of flexible electronic applications, such as sensors and solid electrolytes [13]. By increasing the temperature, the ionic conductivities increased as the ion mobility rose, eventually reaching 1.3 × 10^−3^ S·cm^−1^ and 1.4 × 10^−3^ S·cm^−1^ at 80 °C respectively. The temperature dependence of these ionogels can be described by the Vogel–Tamman–Fulcher (VTF) equation [41,42,43]:

(1) $$\sigma =AT^{-\frac{1}{2}}e^{-\frac{E_{a}}{R\left(T-T_{0}\right)}}$$

where *A* is a temperature-independent constant associated with the number of charge carriers, *E_a_* is the pseudo-activation energy related to polymer segmental motion, and *R* stands for the gas constant. *T*_0_ is a reference temperature usually correlated with the ideal glass transition temperature at which free volume disappears or at which the configurational entropy of the polymer chain reaches zero. In either scenario, *T*_0_ is usually 35 to 50 K below *T_g_* [44,45,46]. The VTF behaviors of all PTE-BDB-IL samples were investigated with *T*_0_ set to *T_g_*—50 K. The values of R^2^ of the VTF linear fit are above 0.99, showing that the VTF model is suitable for describing the ionic behavior of these materials and that polymer chain segmental mobility plays a role in facilitating ion conduction. The VTF of the two samples are very similar, with the parameter *A* found to be 5.6 S·K^1/2^·cm^−1^ for the PTE-BDB25-TFSI50 sample and 5.7 S·K^1/2^·cm^−1^ for the PTE-BDB25-TfO50 sample, and the *E_a_* of ionic conduction of 8.1 kJ·mol^−1^ and 8.5 kJ·mol^−1^ respectively.

**Figure 8:**
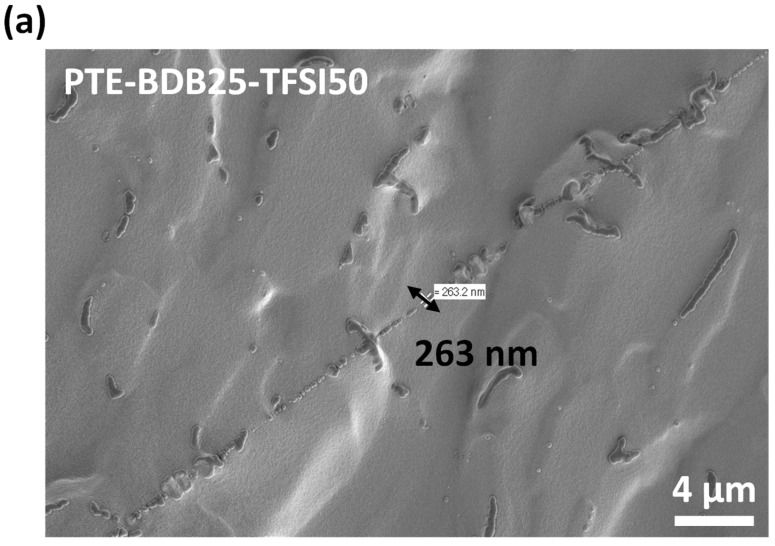

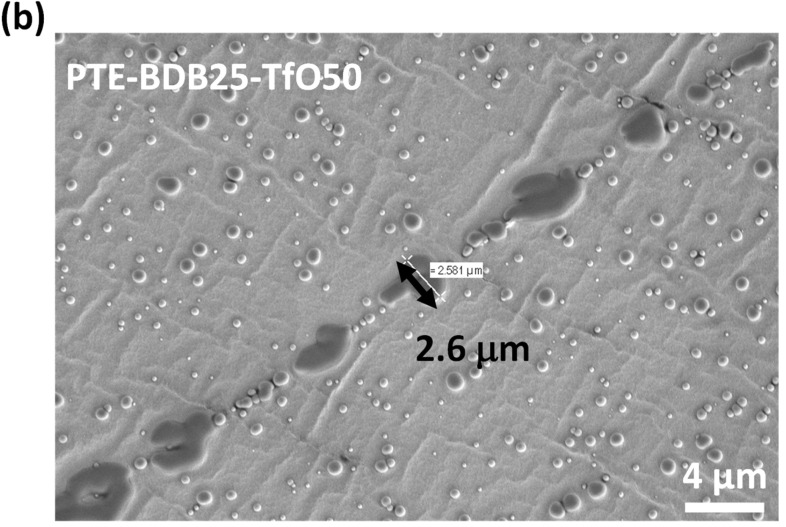
(**a**) SEM image of PTE-BDB25-TFSI50 healed sample cut transection; (**b**) SEM image of PTE-BDB25-TfO50 healed sample cut transection.

PTE-BDB-IL ionogels are also expected to display healing properties because of the dynamic exchange of boronic ester bonds. The two IL-loaded PTE-BDB25 sample films were stacked face-to-face and subjected to a healing experiment at 120 °C for 2 h. For both samples, the two films could not be separated after 2 h, demonstrating healing behavior similar to that of the PTE-BDB25 sample (Figure 7b). To evaluate the healing efficiency of these ionogels, the cross-sections of the stacked samples were examined by scanning electron microscopy (SEM) after healing. A scar of 263 nm between the two stacked films was observed for the PTE-BDB25-TFSI50 sample (Figure 8a), while a slightly larger scar of 2.6 mm was found in the PTE-BDB25-TfO50 sample (Figure 8b). These results demonstrate the healing abilities of these ionogels thanks to the bond exchange reaction, while the remaining scars are consistent with the incomplete relaxation observed at 120 °C. Moreover, the fast relaxation rate in the presence of TFSI^-^ anions may induce a better healing efficiency than triflate anions within the same healing duration. For healable ionogels, the materials must retrieve their ionically conducting behavior after failure. Thus, ionic conductivity measurements were carried out on the samples after the healing experiment. It was found that the healed samples demonstrated similar behaviors as pristine samples, and the ionic conductivity remained in the order of magnitude (Figure 7a). These results indicate that the topology rearrangement of the polymer electrolytes enabled by boronic ester groups allows the materials to heal and recover their original ionically conducting profile.

## 3. Conclusions

In this work, we demonstrated the in-situ preparation of polythioether-based vitrimer ionogels, taking advantage of the thiol-acrylate Michael addition. In the first step, to select the best polymer network of dynamic ionogels, dynamic PTE-based polymer networks were prepared by keeping the amount of trithiol crosslinker constant. The choice of the dithiol spacers varies between a dynamic chain extender BDB containing boronic ester groups (from 0 to 50 mol% of total thiol functions) and static dithiol to control the dynamic properties of these materials, with relaxation times varying with the composition of the samples from 160 min at 60 °C to 36 s at 140 °C. These PTE-BDB networks exhibited vitrimer properties, such as healing and stress relaxation, at elevated temperatures, thanks to the boronic ester exchange reaction. In the second part, dynamic ionogels were prepared using 50 wt% of either EMIM TFSI or EMIM Triflate compared to the total weight. The resulting materials are completely amorphous (*T_g_* around −50 °C), suggesting that these ionogels can be potentially utilized at low temperatures. These ionogels are stretchable with an elongation at break around 60%, soft with Young’s modulus between 0.4 to 0.6 MPa, and demonstrated ionic conductivities in the order of 10^−4^ S·cm^−1^ at room temperature. It has been found that the dynamic properties of these materials, such as stress relaxation (with relaxation time in the same range) and healing, are retained and not significantly modified in the presence of a large quantity of IL. This work further enlarges the library of vitrimer ionogels, and we can envision an easy surface functionalization with either thiol or acrylate groups of these ionogels, thanks to the stoichiometric reactivity of thiol-acrylate Michael addition. The design concept illustrated in this work could potentially open a new path for the development of flexible electrochemical-based electronics with extended service life through repair or reprocessing.

## 4. Materials and Methods

### 4.1. Materials

The photobase generator (PBG) 2-(9-Oxoxanthen-2-yl)propionic acid 1,5,7-triazabicyclo[4.4.0]dec-5-ene salt, 1,4-Butanediol Bis(thioglycolate) (dithiol, DT), and heptane were purchased from TCI Chemicals (Zwijndrecht, Belgium). Poly(ethylene glycol) diacrylate (PEGDA, Mn = 700 g·mol^−1^), trimethylolpropane tris(3-mercoptopropianate) (trithiol, TT), triethylamine, 1-Ethyl-3-methylimidazolium trifluoromethanesulfonate (EMIM Triflate), and 1-thioglycerol were purchased from Sigma-Aldrich (De Schnelldorf, Germany). Magnesium sulfate heptahydrate (MgSO_4_.7H_2_O) was obtained from Acros Organics (Geel, Belgium). Dichloromethane (DCM) was obtained from VMR Chemicals (Fontenay sous Bois, France). 1-Ethyl-3-methylimidazolium bis(trifluoromethylsulfonyl)imide (EMIM TFSI) was purchased from Solvionic (Toulouse, France). Finally, benzene-1,4-diboronic acid was purchased from Apollo Scientific (Stockport, UK).

### 4.2. Synthesis of 2,2′-(1,4-Phenylene)-bis[4-mercaptan-1,3,2-dioxaborolane] (BDB)

The synthesis of dithiol-containing boronic ester 2,2′-(1,4-Phenylene)-bis[4-mercaptan-1,3,2-dioxaborolane] (BDB) was reported by Chen et al. [24]. Benzene-1,4-diboronic acid (3.0 g, 18.1 mmol) and 1-thioglycerol (4.01 g, 37.1 mmol) were dissolved in tetrahydrofuran (80 mL) and water (0.1 mL). Five grams of magnesium sulfate was added to the mixture. After stirring at room temperature for 24 h, the mixture was filtered and concentrated. The resulting solid is purified by repeatedly filtering and washing with abundant heptane, and concentrated to obtain the target compound as white solids (yield 80%). The successful synthesis of BDB was explicitly confirmed by ^1^H NMR (Figure S1). ^1^H NMR (400 MHz, CDCl_3_) δ 7.83 (s, 4H), 4.74 (m, 2H), 4.49 (dd, 2H), 4.18 (dd, 2H), 2.81 (dd, 4H), 1.48 (t, 2H).

### 4.3. Preparation of PTE-BDB Dynamic Networks

In a vial, the dithiol-containing boronic ester (BDB) is solubilized with acetone (m_solvent_ = 0.5′m_PEGDA_) before introducing thiol precursors (TT and/or DT) and the acrylate precursor PEGDA. In parallel, the photobase generator PBG (1 wt% of the total weight of thiol and acrylate precursors) is dissolved in EtOH (50 mg·mL^−1^) and then added into the vial under a light-protected condition. The mixture was poured into a mold consisting of two glass plates separated by a 0.5 mm thick Teflon spacer. Free-standing PTE-BDB films are obtained by curing the precursor solution with a UV curing conveyor system (Primarc UV Technology, Slough, UK, Minicure, Mercury vapor Lamp, UV intensity 100 W·cm^−2^, duration of each scan 4 s). Fifty UV passages were applied for each sample. The acetone was evaporated under vacuum after synthesis at 50 °C for 1 day.

### 4.4. Preparation of PTE-BDB-IL Dynamic Ionogels

In a vial, the dithiol-containing boronic ester (BDB) is solubilized with acetone (m_solvent_ = 0.5′m_PEGDA_) before introducing thiol precursors (TT and/or DT), acrylate precursor PEGDA), and either EMIM TFSI or EMIM Triflate (50 wt% vs total weight). In parallel, the photobase generator PBG (1 wt% of the total weight of thiol and acrylate precursors) is dissolved in EtOH (50 mg·mL^−1^) and then added into the vial under a light-protected condition. The mixture was cast into a mold consisting of two glass plates separated by a 0.5 mm thick Teflon spacer. Free-standing PTE-BDB-IL films are obtained by curing the precursor solution with a UV curing conveyor system after 50 scanning passages (Primarc UV Technology, Minicure, Mercury vapor Lamp, UV intensity 100 W·cm^−2^, duration of each scan 4 s). The acetone was evaporated under vacuum after synthesis at 50 °C for 1 day. The resulting network was named PTE-BDB***X***-TFSI***Y*** or PTE-BDB***X***-TfO***Y*** for a PTE network containing X mol% of BDB and Y wt% of EMIM TFSI or EMIM Triflate.

### 4.5. Methods and Techniques

Nuclear Magnetic Resonance Spectroscopy (NMR): ^1^H NMR spectra were recorded at 297 K on a AVANCE 400 spectrometer (Bruker, Karlsruhe, Germany) at 400 MHz and referenced to the residual solvent peaks (^1^H, δ 7.26 ppm for CDCl_3_).

Infrared spectroscopy (IR): Attenuated total reflection (ATR)-FT-IR spectroscopy was performed using a Tensor 27 (Bruker, Champs-sur-Marne, France) FT-IR instrument equipped with an ATR accessory unit.

Extractable content: Soxhlet experiments were performed with a BUCHI SpeedExtractor E-914 (Villebon sur Yvette, France). The extractable content was determined by 3 cycles of extraction in DCM at 60 °C under 100 bar. Each cycle lasted about 15 min.

Rheology: Rheological measurements were performed with an Anton Paar Physica MCR 301 rheometer (Graz, Austria) equipped with a CTD 450 temperature control device and a plate-plate geometry (Gap 500 μm, diameter 25 mm, plate; polymerization system made from a lower glass plate coupled with a UV lamp 142 mW·cm^−2^). A 1% deformation was imposed at 1 Hz. The storage modulus (G′) and loss modulus (G″) were recorded as a function of time. The solution of precursors of materials was put in the rheometer geometry, and measurements began immediately with UV exposure at 30 °C.

Thermogravimetric analysis (TGA): TGA experiments were performed in air on a Q50 model (TA Instruments, New Castle, DE, USA) applying a heating rate of 10 °C·min^−1^ to 600 °C.

Differential Scanning Calorimetry (DSC): Glass transitions of the materials were determined by DSC. Sequences of temperature ramps (heating, cooling jump, heating, cooling, heating) in the −80 to 180 °C range were performed at 20 °C·min^−1^ ramping up and 5 °C·min^−1^ cooling down using a TA Instruments Q100 model (New Castle, USA) equipped with a liquid cooling accessory and calibrated using sapphire and high purity indium metal. All samples were prepared in hermetically sealed pans (5−10 mg/sample) and were referenced to an empty pan. The reported *T_g_* values were obtained from the second heating cycle.

Tensile testing: Traction experiments were performed on a Dynamic Mechanical Analyzer instrument (TA Instruments, Q800 model, New Castle, USA) in tensile mode at room temperature. A strain rate of 20%·min^−1^ to 500% was applied with an initial strain of 0.05% and a preload force of 0.01 N to obtain stress-strain curves.

Dynamic mechanical analysis (DMA): DMA experiments were conducted on Q800 (TA Instruments, New Castle, USA) in tension mode. Heating ramps were performed from −70 °C to 200 °C at a constant rate of 3 °C·min^−1^ with a maximum strain amplitude of 0.05% at a fixed frequency of 1 Hz, and a preload force of 0.01 N.

Stress relaxation measurement: Stress relaxation measurements were carried out on the Q800 at different temperatures. A preload force of 0.01 N and a constant strain of 3% were applied, and stress decay was monitored over time.

Ionic conductivity: Ionic conductivity was measured by electrochemical impedance spectroscopy using a VSP 150 potentiostat (Biologic SA, Grenoble, France). Samples were placed between two gold electrodes and placed in a thermostated cell under an argon atmosphere. Experiments were carried out in a temperature range from 25 to 100 °C, in the frequency range from 2 MHz to 1 Hz with a rate of 6 points per decade, and for an oscillation potential of 10 mV. The ionic conductivity σ *(S·*cm^−1^) is calculated using Equation (2):

(2) $$\sigma =\frac{1}{Z}\frac{d}{S}\;$$

where Z is the real part of the complex impedance (ohms), d the thickness of the sample (cm), and S is the sample area (cm^2^).

Healing test: Sample films were first cut into two pieces and stacked together. The films were protected by Teflon films, pressured by 2 glass plates with clips, and then heated at 120 °C for 2 h in the oven.

Scanning electronic microscopy (SEM): The samples were mounted directly on SEM stubs, sputtered with 4 nm of platinum (ACE600, Leica, Wetzlar, Germany), and imaged using a Field Emission Gun Scanning Electron Microscope (GeminiSEM300, Carl Zeiss, Oberkochen, Germany) with an acceleration voltage of 2 keV under a high vacuum. Secondary electrons were collected. Scan speed and line averaging were adjusted during the observation.

Calculation of topology freezing temperature *T_v_* and activation energy of the viscous flow *E_a_*: Based on Maxwell’s model for viscoelastic fluids, the stress relaxation behavior of the vitrimer can be described with Equation (3), where the relaxation time *t* is determined as the time required to relax to 37% (1/e) of the initial stress [31]:

(3) $$\frac{\sigma_{\left(t\right)}}{\sigma_{0}}=e^{-\frac{t}{\tau }}$$

For vitrimers, relaxation times reflect associative exchange reactions, and their temperature dependence can be fitted to the Arrhenius equation (Equation (4)) [33,47] :

(4) $$\tau \left(T\right)=\tau_{0}e^{\frac{E_{a}}{RT}}$$

The values of t were then plotted as a function of temperature to determine the activation energy *E_a_* of the associative exchange reaction. The topology freezing temperature *T_v_* is another key characteristic of vitrimer materials. Conventionally, the hypothetical *T_v_* is chosen as the temperature at which the viscosity equals 10^12^ Pa·s as this value describes the liquid-to-solid transition of a glass-forming liquid [14,20]. The relation between viscosity η and the characteristic relaxation time τ can be expressed with the Maxwell relation (Equation (5)) [48]:

(5) $$\eta =G\cdot \tau =\frac{E\text{’}}{2\left(1+\nu \right)}\cdot \tau$$

where *G* stands for the shear modulus, ν for the Poisson’s ratio, and *E*′ for the storage modulus at the rubbery plateau. Using the Poisson’s ratio = 0.5 usually used for rubbers, [33,48] *T_v_* is determined by combining Equations (4) and (5).

## Figures and Tables

**Table 1:** Compositions and characterizations of PTE-BDB networks.

Sample	Functional Groups Molar Ratio (%)				Extractable Content (wt%)	*T_g_* (DSC) (°C)	Young’s Modulus (MPa)	Elongation at Break (%)
	BDB	DT	TT	PEGDA				
PTE-BDB0	0	50	50	100	3.9	−45.8	1.2 ± 0.1	79 ± 20
PTE-BDB25	25	25	50	100	8.0	−41.0	1.5 ± 0.1	76 ± 42
PTE-BDB50	50	0	50	100	12.5	−33.3	1.3 ± 0.1	68 ± 5

**Table 2:** Compositions and details of the PTE-BDB-IL samples.

Sample	IL Type	IL Content (wt%)	Extractable Content (wt%)	*T_g_* (DSC) (°C)	T_α_ (DMA) (°C)	Young’s Modulus (MPa)	Elongation at Break (%)
PTE-BDB25			8	−41.0	−24.2	1.2 ± 0.1	79 ± 20
PTE-BDB25-TFSI50	EMIM TFSI	50	53.8	−49.4	−28.0	0.4 ± 0.1	61 ± 4
PTE-BDB25-TfO50	EMIM Triflate	50	56.3	−51.8	−29.8	0.6 ± 0.1	52 ± 4

## Supplementary Materials

The following supporting information can be downloaded at: https://www.mdpi.com/article/10.3390/gels8060381/s1. Figure S1: ^1^H NMR of 2,2′-(1,4-Phenylene)-bis[4-mercaptan-1,3,2-dioxaborolane] (BDB) in CDCl_3_; Figure S2: ^1^H NMR of the extractable content of PTE-BDB25 sample in CDCl_3_; Figure S3: ^1^H NMR of the extractable content of PTE-BDB50 sample in CDCl_3_.; Figure S4: DSC curves of thermal cured PTE-BDB series samples.; Figure S5: DSC curves of thermal cured PTE-BDB-IL dynamic ionogels and PTE-BDB25 sample. Table S1: Relaxation times extracted from stress relaxation tests of PTE-BDB samples and their corresponding theoretical *T_v_* and *E_a_* associated with the boronic ester exchange reaction.; Table S2: Relaxation times extracted from stress relaxation tests of PTE-BDB-IL samples and their corresponding theoretical *T_v_* and *E_a_* associated with the boronic ester exchange reaction.
