# Supplementary material for: Photopolymerizable Ionogel with Healable Properties Based on Dioxaborolane Vitrimer Chemistry
Source: Gels. 2022 Jun 15;8(6):381. doi: 10.3390/gels8060381 (PMC9222776; doi:10.3390/gels8060381)
Supplement: Supplementary file 1 [file gels-08-00381-s001.zip › gels-1755796-supplementary.pdf]

gels

Supplementary Materials

Article

Photopolymerizable Ionogel with Healable Properties Based on Dioxaborolane Vitrimer Chemistry

Fengdi Li, Giao T. M. Nguyen, Cédric Vancaeyzeele, Frédéric Vidal and Cédric Plesse *

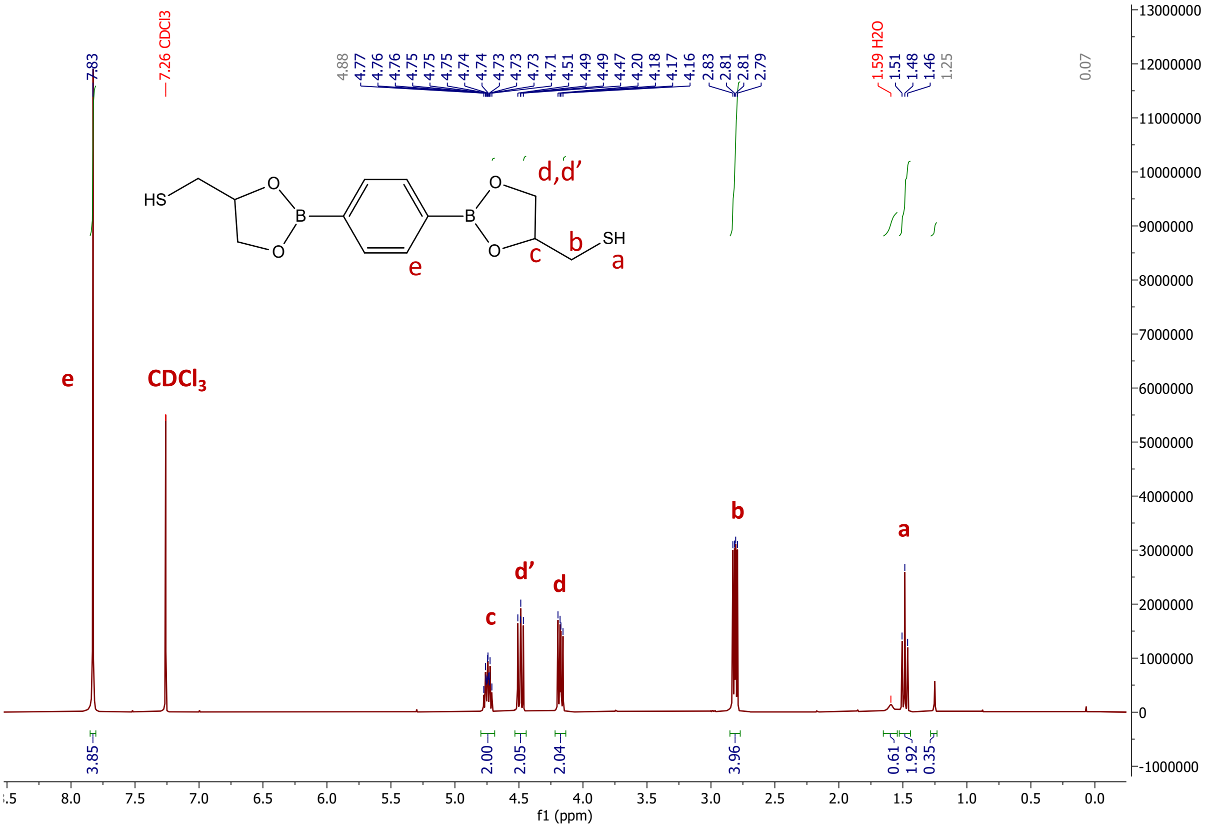

Figure S1. ^{1}H NMR of 2,2′-(1,4-Phenylene)-bis[4-mercaptan-1,3,2-dioxaborolane] (BDB) in $CDCl_3$.

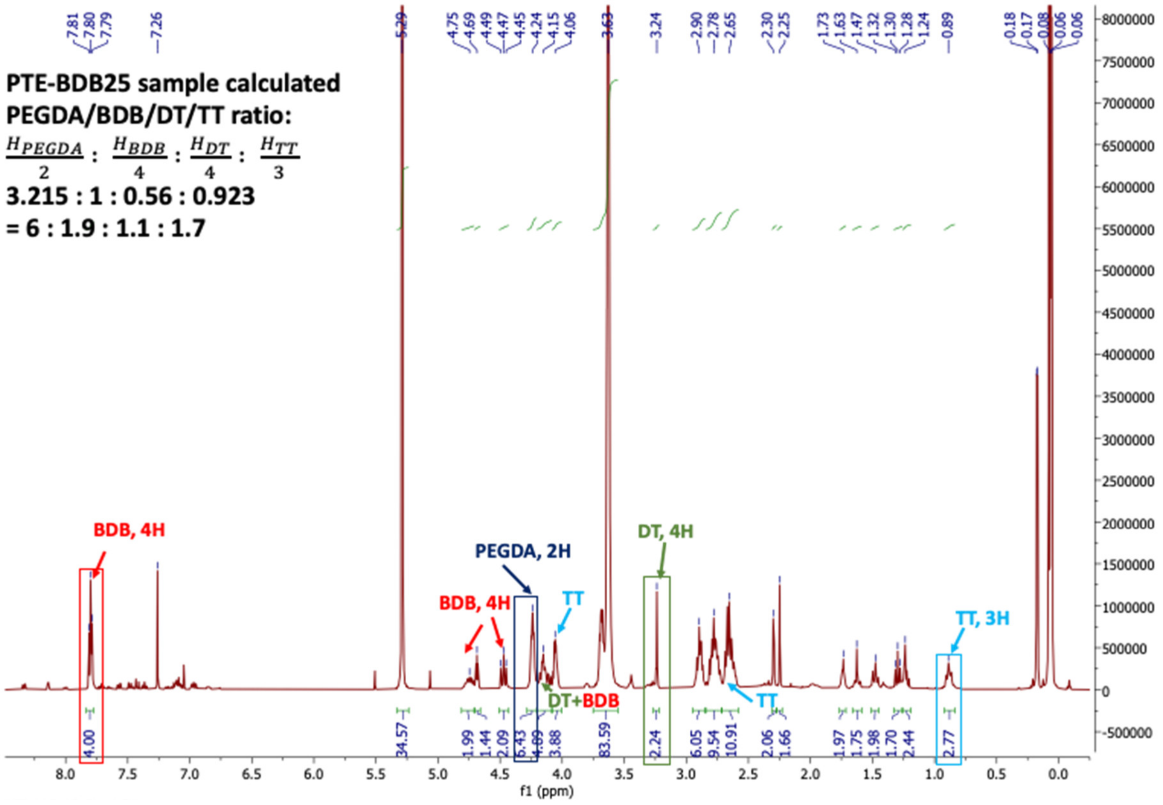

Figure S2. ^{1}H NMR of the extractable content of PTE-BDB25 sample in $CDCl_3$.

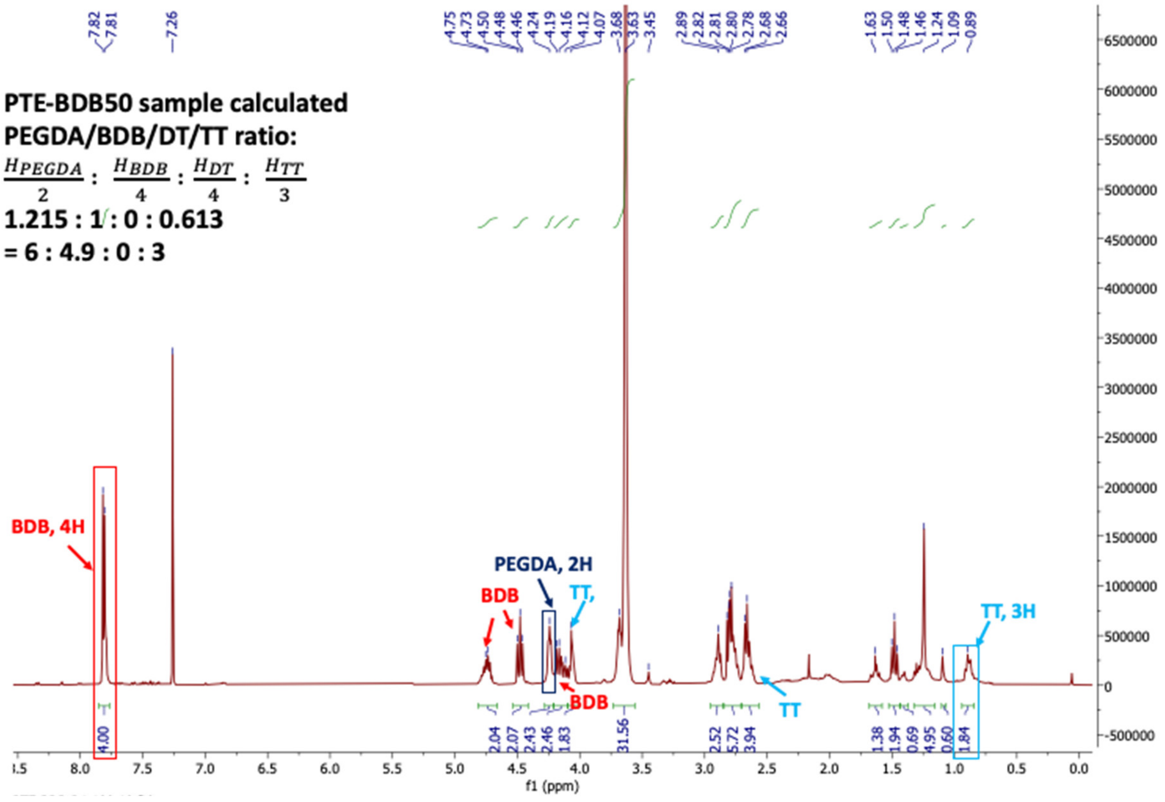

Figure S3. ^{1}**H** NMR of the extractable content of PTE-BDB50 sample in $CDCl_3$.

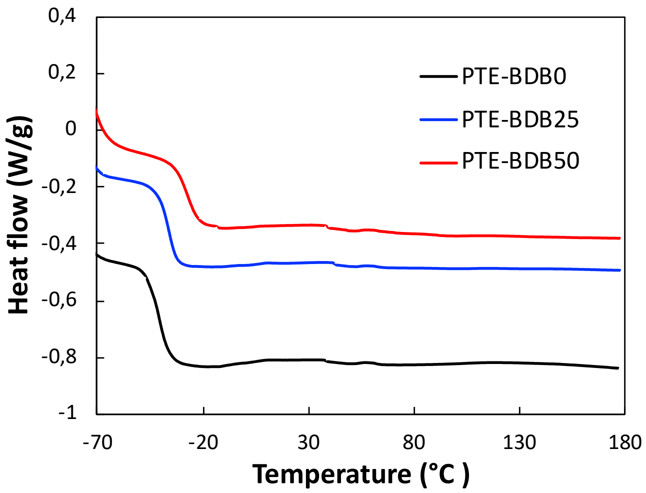

Figure S4. DSC curves of thermal cured PTE-BDB series samples.

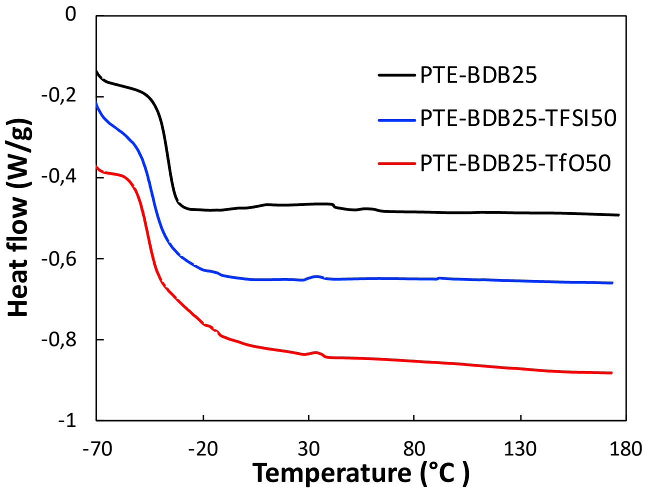

Figure S5. DSC curves of thermal cured PTE-BDB-IL dynamic ionogels and PTE-BDB25 sample.

Table S1. Relaxation times extracted from stress relaxation tests of PTE-BDB samples and their corresponding theoretical T_v and E_a associated with the boronic ester exchange reaction.

Sample	Relaxation time (min)						T_v (°C)	E_a (kJ·mol^{-1})
	60 °C	80 °C	100 °C	120 °C	130 °C	140 °C		
PTE-BDB25	160	53.9	3.5	3.1	1.4	1.1	11.7	75.6
PTE-BDB50	/	10.0	1.3	1.4	/	0.6	-31.0	49.9

Table S2. Relaxation times extracted from stress relaxation tests of PTE-BDB-IL samples and their corresponding theoretical T_v and E_a associated with the boronic ester exchange reaction.

Sample	Relaxation time (min)					Tv (°C)	Ea (kJ·mol^{-1})
	60 °C	80 °C	100 °C	120 °C	140 °C		
PTE-BDB25	160	53.9	3.5	3.1	1.1	11.7	75.6
PTE-BDB25-TFSI50	214	9.4	2.6	1.7	0.6	0.3	78.7
PTE-BDB25-TfO50	211	58.1	8.9	4.1	1.6	2.0	72.0
